# Efficacy of Topical Vancomycin- and Gentamicin-Loaded Calcium Sulfate Beads or Systemic Antibiotics in Eradicating Polymicrobial Biofilms Isolated from Diabetic Foot Infections within an *In Vitro* Wound Model

**DOI:** 10.1128/AAC.02012-20

**Published:** 2021-05-18

**Authors:** G. S. Crowther, N. Callaghan, M. Bayliss, A. Noel, R. Morley, B. Price

**Affiliations:** a Division of Pharmacy and Optometry, Lydia Becker Institution of Immunology and Inflammation, Faculty of Biology Medicine and Health, University of Manchester, Manchester, United Kingdom; b Department of Microbiology, Southmead Hospital, North Bristol NHS Trust, Bristol, United Kingdom; c Podiatric Surgery Department, Buxton Hospital, Derbyshire Community Health Services, NHS Foundation Trust, Buxton, United Kingdom

**Keywords:** biofilm, clinical, diabetes, diabetic foot, infection, polymicrobial, postantibiotic effect

## Abstract

Diabetic foot ulcers are notoriously difficult to heal, with ulcers often becoming chronic, in many cases leading to amputation despite weeks or months of antibiotic therapy in addition to debridement and offloading. Alternative wound biofilm management options, such as topical rather than systemic delivery of antimicrobials, have been investigated by clinicians in order to improve treatment outcomes. Here, we collected blood and tissue from six subjects with diabetic foot infections, measured the concentrations of antibiotics in the samples after treatment, and compared the microbiota within the tissue before treatment and after 7 days of antibiotic therapy. We used an *in vitro* model of polymicrobial biofilm infection inoculated with isolates from the tissue we collected to simulate different methods of antibiotic administration by simulated systemic therapy or topical release from calcium sulfate beads. We saw no difference in biofilm bioburden in the models after simulated systemic therapy (representative of antibiotics used in the clinic), but we did see reductions in bioburden of between 5 and 8 logs in five of the six biofilms that we tested with topical release of antibiotics via calcium sulfate beads. Yeast is insensitive to antibiotics and was a component of the sixth biofilm. These data support further studies of the topical release of antibiotics from calcium sulfate beads in diabetic foot infections to combat the aggregate issues of infectious organisms taking the biofilm mode of growth, compromised immune involvement, and poor systemic delivery of antibiotics via the bloodstream to the site of infection in patients with diabetes.

## INTRODUCTION

Diabetes mellitus is a major public health concern with an estimated prevalence of 451 million people living with the condition in 2017 (1). This figure is estimated to rise to 693 million people by 2045. A frequent and serious complication of diabetes mellitus is the development of diabetic foot ulcers (DFUs), which effects an estimated 25% of those with diabetes within their lifetime (2) and costs the UK National Health Service between £524.6 and £728 million per annum (3). Structural foot abnormalities, sensory neuropathy, and peripheral arterial disease are leading risk factors for the onset of DFU formation. Up to 60% of foot ulcers display signs of clinical infection at initial presentation at a diabetic foot clinic, resulting in increased health care costs and risk of limb loss (3). Successful treatment of diabetic foot infections (DFIs) is poor, with a 1-year observational study reporting a 44.5% incidence of unhealed DFIs (4). For these patients, 23.4% required revascularization or amputation, 4.3% exhibited ulcer recurrence, and there was a 15.1% patient death rate. Treatment of diabetic foot infection is often protracted and ranges from combinations of oral antibiotics, intravenous (i.v.) antibiotics, wound debridement, and amputation (5).

The severity and chronicity of a DFI is dependent on a number of factors, including the level of host immune response, the bacterial load, and the virulence of the microorganisms present (6). Many commensal microorganisms exist on the surface of the skin and within the DFU (7). These microorganisms often cause no harm, and some are even beneficial, providing colonization resistance against the invasion of pathogenic microorganisms. Numerous microorganisms have been associated with DFIs, but their relative contribution to symptomatic and chronic infection remains unknown (8–10).

The ability of wound isolates to grow within biofilms is well documented, with polymicrobial biofilms present in up to 78% of DFIs (11–14). The capacity of microorganisms to form these complex biofilm structures is associated with exacerbated chronicity, owing in part to impaired wound healing and the recalcitrance of sessile organisms to the innate immune response and standard antimicrobial therapy (15).

Peripheral arterial disease (PAD) is a causative factor in approximately 50% of all DFUs and leads to chronic ulcer presentation and thus further increased risk of ulcer infection (16, 17). Moreover, PAD leads to decreased blood flow to the extremities, particularly the distal limb, and therefore the site of infection, in individuals with diabetes. Current treatment guidelines for clinically infected DFUs recommends debridement, which is an important strategy for disrupting biofilm (18), followed by the administration of systemic antimicrobial therapy: flucloxacillin in the first instance or, alternatively, clarithromycin, erythromycin, or doxycycline in the case of penicillin allergy in combination with patient offloading (5, 19). Due to the combined effects of reduced blood flow to the site of infection as a result of PAD, the presence of biofilm communities in DFIs, and the increased antimicrobial levels required to eradicate biofilm infection, successful treatment of DFIs is uncommon (20–23), and therefore other treatment options have been sought (24, 25). There is some evidence that topical antibiotic agents in the form of dressings may improve healing of DFIs (26) and that there is no additional risk of adverse events when comparing topical treatments to systemic antibiotics; however, more work needs to be done in this area (26). Moreover, there is some evidence that the use of topical antibiotics reduces surgical site infection risk (27). Risks associated with topical antibiotics include systemic toxicity; one case study has shown vancomycin toxicity with cobalt cement (28), and another has shown vancomycin toxicity with cement spacers (29). However, observational and case studies have not found systemic toxicity from vancomycin-loaded calcium sulfate (CS) void fillers (23, 30), which have been used safely as a delivery vehicle for the local release of antibiotics (31) and have shown efficacy against biofilms in previous *in vitro* studies (25, 32, 33), although further work is required to demonstrate efficacy in a clinical setting.

In order to investigate novel treatment approaches to DFI, *in vitro* modeling is required in the first instance, particularly in the case of biofilms (34). There are many models available with different strengths and weaknesses, and they tend to be useful for different scenarios such as chronic versus acute infection or high- versus low-exudate wounds (35). Few models incorporate polymicrobial biofilms or clinical isolates (11, 12). We have previously developed and validated a clinically relevant three-dimensional collagen biofilm model to investigate the efficacy of antibiotic-loaded CS beads in eradicating monomicrobial and polymicrobial biofilms (36, 37). In the present study, the overall aim was to compare the antibiofilm efficacy of a novel topical antibiotic combination and standard systemic antibiotics using our *in vitro* model of biofilm infection. To this end, we completed a study composed of three parts. (i) We collected tissue samples and blood samples from patients. This allowed us to establish clinically relevant concentrations of antibiotics in blood and tissue and determine conditions to test *in vitro*. (ii) Next, we collected and catalogued clinical isolates from diabetic foot tissue that had interacted *in vivo* and therefore were more likely to form polymicrobial biofilms in coculture *in vitro*. In parallel, we gathered data on the impact of systemic therapy on the *ex vivo* microbiota composition and susceptibility to antibiotics for comparison to our wound models. (iii) Finally, we simulated the *in vivo* polymicrobial biofilms using our *in vitro* models and compared the efficacy of simulated local antibiotics via vancomycin and gentamicin-loaded CS beads to systemic antimicrobial therapy in eradicating the resultant clinically relevant biofilms. The MICs were to the vancomycin-gentamicin combination were determined to better understand the log reductions seen in the *in vitro* modeling. Our results are split into corresponding subsections to reflect these parts of the study.

## RESULTS

### Serum and tissue antibiotic levels.

Six patients (DFG, DFN, DFR, DFK, DFB, and DFR) with DFIs were successfully recruited for the study. Data on sex, age, obesity, peripheral vascular disease (PVD), and neuropathy status, as well as kidney function and wound grade at the time of recruitment, are shown in Table 1. Debrided tissue from each ulcer was collected upon presentation (day 0) and after a week of treatment (day 7). Data on the systemic antibiotics chosen for treatment was recorded and blood samples were collected at day 7. Serum and tissue samples were assayed for the antibiotics used in treatment (Table 2). Patients DFG, DFN, and DFR were prescribed combinations of antibiotics (Table 2). We were able to assay only one of the two antibiotics for patients DFG and DFN. Ciprofloxacin was detectable in the serum but was below the lower limits of quantification (LLOQ) in tissue for patient DFG. Amoxicillin was below the LLOQ in all samples for patient DFN. Trimethoprim and sulfamethoxazole were assayed in serum for patient DFR, but both antibiotics were below the LLOQ for the assay in tissue.

**TABLE 1:** Patient demographic data^*a*^

Patient	Sex	Age	Obese	PVD	Neuropathy	Renal function stage	Wound grade
DFG	M	55	Y	N	Y	CKD 3a	B1
DFN	M	69	Y	Y	Y	CKD 3a	D3
DFR	F	67	Y	N	Y	CKD 3a	B1
DFB	F	52	Y	N	Y	CKD 3a	B2
DFK	M	78	N	N	Y	CKD 3b	B1
DFM	M	65	Y	N	Y	CKD 3a	B3

a “M” or “F” indicates male or female; “Y” or “N” indicates yes or no. PVD was defined as the presence or absence of palpable pedal pulses. CKD, chronic kidney disease. All data were accurate at time of infection. Wounds are graded based on the University of Texas classification system (50).

**TABLE 2:** Oral antibiotic dosing information and posttreatment serum and tissue antibiotic concentrations for subjects recruited on this study^*a*^

Patient	Antibiotic therapy	Dose regimen	Last dose (h)	Antibiotic, concn (mg/liter) in:	
				Serum	Tissue
DFG	Ciprofloxacin	500 mg BD	3	1.3	<0.2
Clindamycin	450 mg QDS	ND	ND
DFN	Co-amoxiclav	625 mg TDS	17	Amoxicillin, <1.0	Amoxicillin, <1.0
Clavulanic acid, ND	Clavulanic acid, ND
DFR	Co-trimoxazole	960 mg BD	42	Trimethoprim, 1.1	Trimethoprim, <1.0
Sulfamethoxazole, 6.9	Sulfamethoxazole, <1.0
DFB	Flucloxacillin	1 g QDS	7.5	0.4	<0.1
DFK	Flucloxacillin	1 g QDS	1	11.5	0.25
DFM	Flucloxacillin	1 g QDS	4	7.84	0.453

a The last dose refers to the number of hours between the last dose of antibiotics taken within the course and the time at which samples were taken. Assays for the quantification of clavulanic acid and clindamycin were not available. Therefore, data for these antibiotics were not determined (ND). Patients were prescribed antibiotics on day 0 of treatment, upon presentation with an ulcer, and samples were taken after a week of treatment at the next diabetic foot clinic. All patients reported adherence to the course of antibiotics. BD, twice daily; TDS, three times daily; QDS, four times daily.

Patients DFB, DFK, and DFM were prescribed flucloxacillin, with concentrations between 0.4 and 11.5 mg/liter present in serum. Flucloxacillin was below the LLOQ in tissue samples from subject DFB, but 0.25 to 0.45 mg/liter was detected in tissue in subjects DFK and DFM.

### Characterization of microbiota in debrided tissue samples. (i) Microbiota isolated from tissue samples of DFIs.

**FIG 1:**
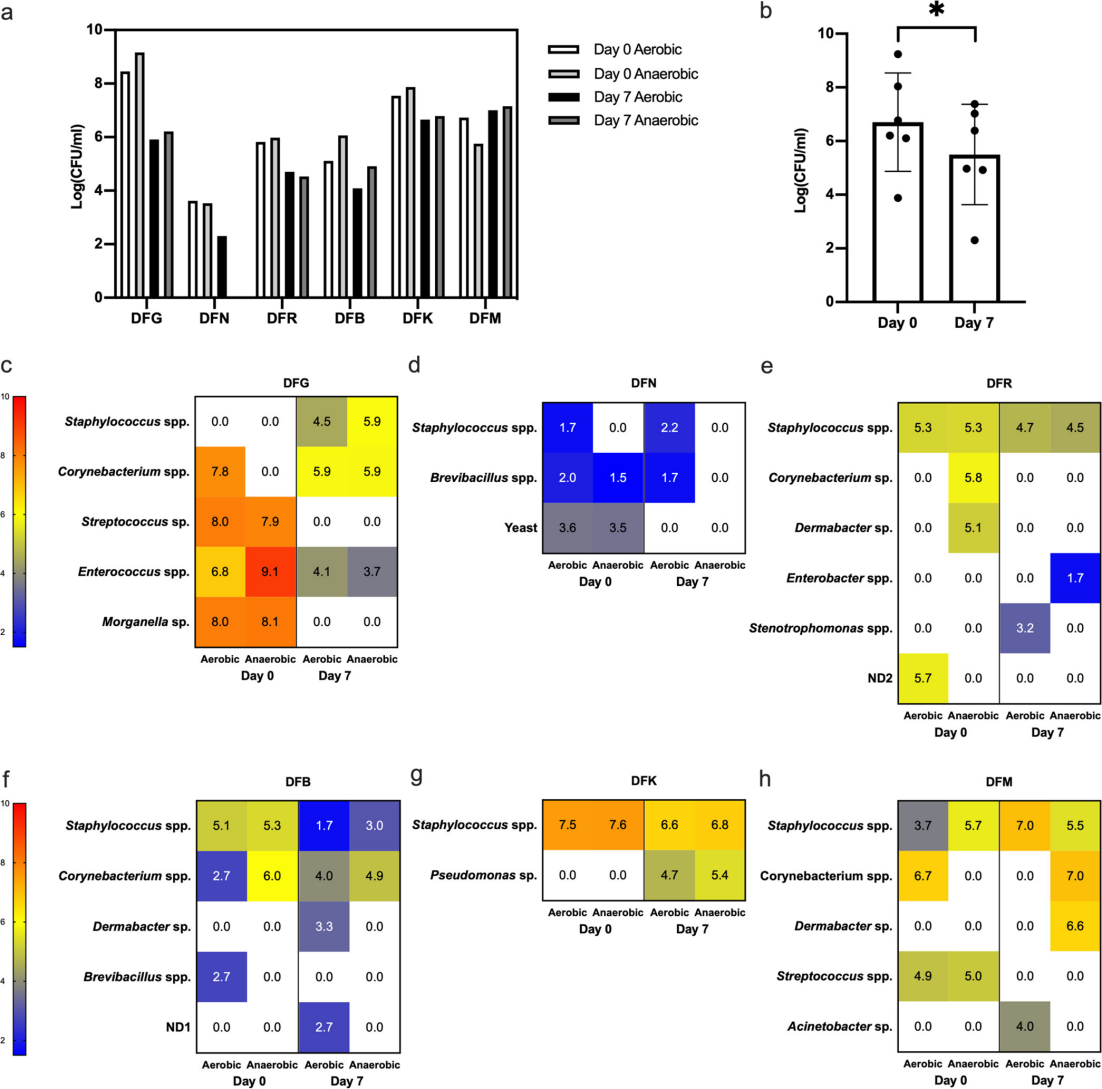
Microbiota from tissue samples of DFIs for six subjects. (a) Total counts of aerobic and facultative anaerobic microorganisms isolated from debrided tissue from patients pre- and posttreatment (systemic) for a diabetic foot infection. (b) Average combined total counts ± the standard deviations (SD) for all patients pre- and posttreatment. (c to h) Heat maps showing the change in microbiota groups isolated from debrided tissue under aerobic or anaerobic growth conditions from each patient upon presentation and after 7 days of systemic treatment with antibiotics for a diabetic foot infection. The limit of detection for each species was 1.7 log_10_(CFU/ml) in this study. ND1 and ND2 refer to two strains that we were unable to identify.

The microbiota recovered from tissue samples pre- and posttreatment were enumerated and identified. The total counts of aerobic and facultative anaerobes for all six patients are presented in Fig. 1a. Counts between subjects’ DFIs were extremely varied, ranging between 3.9 and 9.2 log_10_(CFU/ml) at day 0 and 2.3 to 7.4 log_10_(CFU/ml) at day 7. In five of six subjects, the total number of aerobic organisms present within debrided tissue posttreatment were lower than those pretreatment [range, 0.9 to 2.5_10_(CFU/ml) lower], except for subject DFM, where counts increased by 0.3 log_10_(CFU/ml). The total number of facultative anaerobic organisms present within debrided tissue posttreatment were lower than those pretreatment [range, 1.1 to 3.5 log_10_(CFU/ml) lower], with the exception of the same subject, DFM, for whom counts increased by 1.4 log_10_(CFU/ml).

Total counts for all patients pre- and posttreatment decreased from an average of 6.7 log_10_(CFU/ml) pretreatment to 5.5 log_10_(CFU/ml) posttreatment (Fig. 1b). This reduction of 1.2 log_10_(CFU/ml) was statistically significant (*P* < 0.05).

The composition of microbiota isolated from the tissue of patients pre- and posttreatment is summarized in Fig. 1c to h. *Staphylococcus* species were present in five of the six subjects upon presentation with an infected ulcer and in all of the patients after a week of treatment. In subject DFG, no *Staphylococcus* was detected before treatment, the most abundant species of bacteria present were *Streptococcus* sp. and *Morganella* sp. These organisms were not present after dual therapy of ciprofloxacin and clindamycin; however, *Staphylococcus* spp. had now become part of the ulcer microbiota.

*Corynebacterium* spp. were the next most prevalent organism in tissue samples, followed by *Dermabacter* spp. (Fig. 1c to h). In addition to bacterial species, yeast species were also identified on day 0 in patient DFN but not on day 7. In patient DFK there was a small decrease in abundance of *Staphylococcus* spp. after treatment with flucloxacillin; however, *Pseudomonas* sp., which is not susceptible to flucloxacillin, became prevalent. In DFM, the only patient in whom the counts increased from day 0 to day 7, *Streptococcus* present at day 0 was not detected at day 7. However, *Dermabacter* and Acinetobacter were detected at day 7 but not at day 0.

### (ii) MICs of bacteria isolated from debrided tissue to antibiotics used in treatment.

The MICs of antibiotics used in the management of patients against isolates recovered from corresponding debrided tissue are summarized in Table 3. For subjects DFG, DFN, and DFR, the serum concentration of antibiotics was lower than the experimentally determined MIC in most cases. However, these data are incomplete and complicated by dual therapy. For subjects DFR, DFB, and DFK, who were treated with flucloxacillin, of the six strains for which an MIC was established from day 0, all had MICs below the concentration of flucloxacillin assayed in the serum after a week of treatment (see Table 2 compared to Table 3). However, in samples from day 7, four of the six species were again present, and three of these had an increased MIC. Five additional strains were detected at day 7 compared to day 0; of these strains, four were resistant to flucloxacillin (MIC >16).

**TABLE 3:** MIC (mg/liter) ranges of microorganisms isolated from diabetic foot infections

Patient (treatment)	Isolate	MIC to treatment antibiotic (mg/liter)^*a*^	
		Pretreatment	Posttreatment
DFG (ciprofloxacin-clindamycin)	*Staphylococcus* spp.	–	0.5–4
*Corynebacterium* spp.	16	32
*Streptococcus* sp.	4	–
*Enterococcus* spp.	4	4
*Morganella* sp.	0.25	–
DFN (amoxicillin-clavulanic acid)	*Staphylococcus* spp.	2	0.25–1
*Brevibacillus* spp.	0.5	0.5
Yeast	>128	–
DFR (trimethoprim-sulfamethoxazole)	*Staphylococcus* spp.	8	4–>256
Corynebacterium sp.	ND	–
*Dermabacter* sp.	>256	–
*Enterobacter* spp.	–	2
*Stenotrophomonas* spp.	–	ND
ND2	>256	–
DFB (flucloxacillin)	*Staphylococcus* spp.	0.125	0.25–2
*Corynebacterium* spp.	ND	2–8
*Dermabacter* sp.	–	2
*Brevibacillus* spp.	0.125	–
ND1	–	>16
DFK (flucloxacillin)	*Staphylococcus* spp.	0.25	0.25
*Pseudomonas* sp.	–	>16
DFM (flucloxacillin)	*Staphylococcus* spp.	0.125–0.25	0.125–2
*Corynebacterium* spp.	0.125	2
*Dermabacter* sp.	–	>16
*Streptococcus* spp.	1	–
*Acinetobacter* sp.	–	128

a –, the species was not present in either the pre- or the posttreatment sample for each subject. ND, the MIC was not determined because the strain was unable to grow in the test medium.

### *In vitro* assessment of the effect of calcium sulfate beads loaded with vancomycin and gentamicin on polymicrobial biofilms corresponding to each patient at day 0.

Above, we determined the amount of the antibiotic in the tissue and serum of six patients, and then we established the microbial load and composition of debrided tissue from DFIs before and after treatment with oral antibiotics. We also tested the susceptibility of the microbes isolated to those antibiotics used in treatment. We went on to investigate whether we could replicate similar results by modeling biofilms composed of the same strains of bacteria in the laboratory and whether a different method of administering antibiotics could be more effective in reaching supra-MICs of antibiotics.

**FIG 2:**
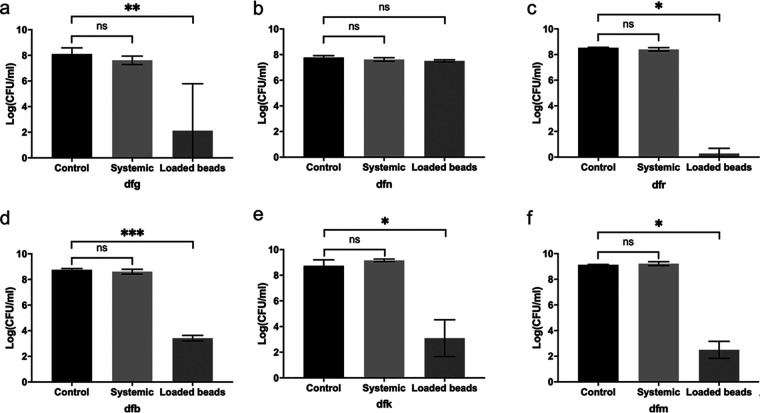
Mean total bacterial counts ± the SD within the wound model after exposure to topical therapy, simulated systemic therapy, or no antibiotics. “Control” denotes models to which no antibiotics were added. (a to f) The model designations (labeled in lowercase letters) correspond to patients DFG, DFN, DFR, DFB, DFK, and DFM discussed in the text. *, *P* < 0.05; **, *P* < 0.01; ***, *P* < 0.001; ns, not significant.

The efficacy of CS beads (Stimulan Rapid Cure [SRC]) loaded with vancomycin and gentamicin has been reported in several case studies (23). In order to evaluate their efficacy in our *in vitro* model, the microbiota isolated from the infected tissues of patients prior to treatment were inoculated into collagen wound models situated in tissue culture inserts within well plates. These polymicrobial biofilms developed in the wound models are labeled in the lowercase letters in Fig. 2 corresponding to each patient (i.e., dfg, dfn, dfk, dfb, dfr, and dfm). Biofilms of these microorganisms were allowed to develop over 3 days before exposure to different treatment conditions, including (i) simulated systemic antibiotics matching the serum concentration established in blood samples, or reported *C*_max_ values where these data were unavailable, (ii) SRC beads loaded with vancomycin and gentamicin placed topically in the wound model, or (iii) no antibiotic treatment (Table 4). Systemic antibiotic delivery was simulated by introduction with media changes, whereas topical antibiotics were introduced in the form of vancomycin- and gentamicin-loaded SRC placed in a void on the surface of the gel for 3 days. After incubation with antibiotics, the bacteria were enumerated (Fig. 2).

**TABLE 4:** Topical and systemic antibiotic dosing levels used in the collagen wound models^*a*^

Patient	Antibiotic	Concn (mg/liter) added to wound model for systemic therapy	Antibiotic (mg) loaded into beads
DFG	Ciprofloxacin	2.4*
Clindamycin	4.2*
DFN	Co-amoxiclav	Amoxicillin, 7.2*
Clavulanic acid, 2.4*
DFR	Co-trimoxazole	Trimethoprim, 1.1	Vancomycin, 500
Sulfamethoxazole, 6.9	Gentamicin, 240
DFB	Flucloxacillin	0.4	Per 10 ml of calcium sulfate
DFK	Flucloxacillin	11.5
DFM	Flucloxacillin	7.8

a When patient serum antibiotic levels were available, these values were used in dosing the models. *, when patient data were not available (i.e., clindamycin and clavulanic acid), *C*_max_ values from the literature (38, 50) for both antibiotics were used.

Biofilms of isolates from pretreatment tissue samples grew to 7.8 to 9.8 log_10_(CFU/ml) in controls to which no antibiotics had been added. Simulated systemic antimicrobial therapy did not significantly reduce biofilm bioburden in any of the wound models; the average log reduction was 0.1 log_10_(CFU/ml), with log reductions ranging from −0.43 to 1.4 log_10_(CFU/ml) (Fig. 2). In models representing subjects DFK and DFM, the total biofilm bioburdens after exposure to systemic antimicrobial therapy increased by 0.4 and 0.1 log_10_(CFU/ml), respectively, relative to that observed in the no-antimicrobial control (Fig. 2). Vancomycin- and gentamicin-loaded SRC significantly reduced the biofilm bioburden in 5 of 6 wound models (Fig. 2). These log reductions ranged from 5.3 to 8.2 log_10_(CFU/ml). Neither vancomycin- and gentamicin-loaded SRC nor simulated systemic antimicrobial therapy reduced the biofilm bioburden in the model representative of subject DFN, likely because of the presence of yeast. Vancomycin-gentamicin SRC beads reduced the biofilm bioburden in model dfr to around the limit of detection [0.57 log_10_(CFU/ml)], which was equivalent to a log reduction of 8.2 log_10_(CFU/ml) (Fig. 2).

### MICs of bacteria isolated from debrided tissue to antibiotics loaded into CS beads.

MICs to the combination of vancomycin and gentamicin in the same ratio as was loaded into SRC beads were undertaken to test the susceptibility of the isolates to these antibiotics (Table 5). In general, the MIC data are consistent with the log reductions observed in the *in vitro* models; DFN had no reduction in bioburden after exposure to antibiotics, and the overall MIC for the combination of bacteria isolated from this patient was high, or in the case of yeast, resistant. Similarly, in microbes isolated from subject DFR the MIC range was between 4 and 8 (although no MIC was determined for the *Corynebacterium* spp.), and the resultant biofilm was almost eradicated in the models. An intermediate MIC of 8 to 32 corresponded to 5.3- to 6.6-log_10_(CFU/ml) reductions in the models dfg, dfb, dfk, and dfm representing subjects DFG, DFB, DFK, and DFM.

**TABLE 5:** MIC ranges of vancomycin/gentamicin against microorganisms isolated from diabetic foot infections at day 0^*a*^

Patient	Isolate	MIC (mg/liter)
DFG	*Corynebacterium* spp.	4
*Streptococcus* sp.	16
*Enterococcus* spp.	16
*Morganella* sp.	32
DFN	*Staphylococcus* spp.	16
*Brevibacillus* spp.	ND
Yeast	>128
DFR	*Staphylococcus* spp.	8
*Corynebacterium* sp.	ND
*Dermabacter* sp.	8
ND2	4
DFB	*Staphylococcus* spp.	1–8
*Corynebacterium* spp.	8
*Brevibacillus* spp.	ND
DFK	*Staphylococcus* spp.	1−16
DFM	*Staphylococcus* spp.	4−16
Corynebacterium spp.	ND
*Streptococcus* spp.	4

a ND, the MIC was not determined because the strain was unable to grow in the test medium.

## DISCUSSION

Diabetic foot infections present significant therapeutic challenges. Standard clinical practice guidelines recommend systemic antimicrobial therapy for clinically infected DFUs with flucloxacillin as the first choice antibiotic for mild or moderate infections (38). However, systemic antibiotics are often ineffective in resolving infection, and chronic recurrent infections are common (39). Peripheral arterial disease in this patient cohort and the presence of recalcitrant biofilm structures within the ulcers pose enhanced challenges in the delivery of effective concentrations of systemic antimicrobial agents to the site of infection. The use of high-dose, long-term, antimicrobial therapy is common in an attempt to combat chronic DFI (40). However, such extreme dosing regimens have been associated with severe adverse effects, including systemic toxicity. The ability to provide antimicrobial therapy at effective concentrations is a key goal for successful treatment outcomes. The use of topical antimicrobial therapies in addition to systemic antimicrobial strategies have previously been used with some success (23, 41).

Here, we harvested debrided tissues from six DFI patients before and after standard oral antimicrobial therapy and determined their microbial compositions. All DFI tissue samples were polymicrobial, with the exception of that from patient DFK, where only *Staphylococcus* spp. were present in the DFI pretreatment. The predominant microorganisms across all patients present prior to treatment were *Staphylococcus* and *Corynebacterium* spp. These data are in accordance with other studies (6, 7, 9). In one study of 454 tissue samples from 433 patients with DFI, the microbiotas of pretreatment samples were determined. Of the positive cultures, 83.8% were polymicrobial, with an average of 2.7 organisms per culture. The predominant organisms were *Staphylococcus* spp., *Streptococcus*, *Enterococcus* spp., *Corynebacterium* spp., *Enterobacteriaceae*, and *Pseudomonas* spp. (42). Although bacteria are usually the organism of focus in chronic wounds, fungal species such as yeasts are also known to exist and contribute toward such infections (11, 12, 43).

DFI microbiota are comprised of a diverse consortium of microorganisms. Interactions between microorganisms are complex and contribute to the virulence, chronicity, and healing of wounds, as well as susceptibility to antibiotics. Synergistic relationships between certain species are often associated with increased virulence and recalcitrant infections with poor clinical outcome (12, 44, 45). For example, previous work has found increased biofilm production when *Staphylococcus* and *Corynebacterium* were cocultured (44); we found this combination of species in four of six tissue samples. Increased tolerance to antibiotics has been reported when *Pseudomonas* and *Staphylococcus* were components of a polymicrobial biofilm (45), a combination that we found in the tissue sample of patient DFK after treatment. The bioburden for this patient after treatment was higher than in the sample taken before treatment. We also saw increases in bioburden in patient DFM from 7.7 to 8 log_10_(CFU/ml) for aerobes and from 6.7 to 8.1 log_10_(CFU/ml) for facultative anaerobes. Corresponding changes in the microbiota demonstrated that the *Streptococcus* in tissue at day 0 was, in effect, replaced by Acinetobacter and *Dermabacter* at day 7. In this subject, the flucloxacillin concentration was high compared to other subjects at 8 mg/liter in blood and 0.5 mg/liter in the tissue, possibly reflecting samples being taken 4 h after the patient took the antibiotics. Interestingly, the MIC data show an MIC for *Streptococcus* of 1 mg/liter compared to MICs of 128 mg/liter for Acinetobacter and 2 mg/liter for *Dermabacter*. Taken together, these data indicate that flucloxacillin was effective in removing the arguably more virulent *Streptococcus* in the day 0 wound, but this was replaced by organisms that were less susceptible to the antibiotics used in treatment. Alterations in the wound microbiota have been associated with better healing (46).

One of the main associated comorbidities of diabetes is peripheral arterial disease which limits blood to the extremities. As a result, we hypothesized that the concentration of oral antimicrobial therapy reaching the site of infection would be low. The concentration of antibiotics in tissue samples was too low to detect in five of seven cases; however, pairwise comparisons between blood and tissue samples taken at a single time point are inappropriate because of the effect of hysteresis (47). A better method to assess tissue penetration of antibiotics is microdialysis. Previous studies have been used to assess the tissue penetration of telithromycin, daptomycin, and linezolid (48). A future study in which microdialysis is used to sample tissue fluid for subsequent assay of first-line antibiotics in DFI, such as flucloxacillin, would provide an accurate representation of the penetration of antibiotics into tissue.

The concentration of antibiotics available at the site of infection in tissue at the time of sampling were sub-MIC for the majority of bacteria isolated (Tables 2 and 3). This is consistent with microorganism counts pretreatment being reduced by an average of only 1.1 log_10_(CFU/ml) after oral antimicrobial therapy. However, in the context of a DFI even a relatively small reduction in the bacterial burden or change in the microbes present in the wound may be sufficient to allow the immune system to control the infection and thereafter reduce inflammation to allow healing to take place (8, 26).

The *in vitro* biofilm models used here incorporate components of the dermal matrix and, in previous studies, have been shown to support robust biofilms that produce extracellular matrix, have increased tolerance to antibiotics, and form microcolonies comparable to those observed in *ex vivo* samples 72 h after inoculation (36, 37, 49). The biofilm bioburden in these models was higher than the bioburden assayed in the tissue before treatment by 2 to 3 log_10_(CFU/ml). This is likely to be a result of increased nutrient availability in the models, as well as a lack of immune involvement, which resulted in a more robust biofilm. No reduction in *in vitro* biofilms was observed after simulated systemic therapy. Moreover, the systemic antimicrobial levels used in the models were equivalent to patient serum levels or *C*_max_ values obtained from the literature rather than those observed in the tissue. Substantial log reductions were elicited when biofilms *in vitro* were exposed to vancomycin- and gentamicin-loaded SRC. Furthermore, MICs are associated with planktonic bacteria, the bacteria present within the model are known to exist in biofilms (36, 37), and antimicrobial concentrations required to eradicate biofilms are substantially higher than those required to eradicate planktonic organisms (22, 36, 37).

The use of local antibiotic carriers, such as CS beads, has the potential to facilitate the release of high antimicrobial levels to the site of infection. The CS beads utilized here contained a combination of gentamicin (240 mg) and vancomycin (500 mg) per 10 ml of SRC. These high antimicrobial levels are more likely to impact biofilms. The combination of gentamicin and vancomycin ensure a spectrum of activity against a wider range of bacteria. Vancomycin, a glycopeptide, demonstrates bactericidal activity against aerobic and anaerobic Gram-positive organisms. It is often used in combination therapy in order to broaden activity, to increase synergy, and to reduce the development of antimicrobial resistance (5). Gentamicin, an aminoglycoside, demonstrates bactericidal activity against a wide range of Gram-negative and some Gram-positive organisms. Synergy between vancomycin and gentamicin has been reported against methicillin-resistant Staphylococcus aureus (30, 34).

Vancomycin- and gentamicin-loaded SRC beads were able to elicit a 2.5- to 8.2-log_10_CFU/ml reduction in five of six patients. In patient DFN, only a 0.3-log_10_CFU/ml reduction was observed in response to vancomycin/gentamicin-loaded CS beads. The microbiota of patient DFN was comprised primarily of yeast. Vancomycin and gentamicin do not possess anti-fungal activity and therefore will have no effect upon yeast present within the wound model for this patient, thereby resulting in the persistence of these microorganism despite exposure to high levels of vancomycin and gentamicin. The 0.3-log_10_CFU/ml reduction observed in the model was likely due to the eradication of the minority of susceptible bacteria present within this patient sample. Interestingly, the 7-day tissue sample from this patient did not contain yeast. This could be because the yeast was removed by debridement or it could reflect a difference in sampling.

### Limitations.

In this study, debrided tissue was collected by the hospital microbiology labs, in addition to our research group, so that there was no interference in normal treatment. This meant that the tissue sample was small, and we could not control the timing of the last dose of antibiotics and of the blood and tissue sampling. Wounds have been shown to be heterogenous in microbial composition (7), and the isolates that we recovered may not have reflected the microbiome of the ulcer in its entirety. Ideally, tissue samples would be taken from different areas of the wound.

The biofilm in these *in vitro* models is 72 h old, and *in vivo* the biofilms could have been established over a longer time period. However, the biofilms within the models showed a smaller difference in log reductions than the *ex vivo* samples, indicating that these biofilms were more robust than those in the tissue.

The number of available results in the antibiotics assayed in *ex vivo* samples is small. This is because of the small number of subjects recruited for this study within the 12-month time frame of the ethics and the diversity in treatments used for those subjects, making it difficult to assay the various antibiotic therapies. This has resulted in some cases in the *C*_max_ reported in the literature being used instead of empirical data. Further, the concentration of antibiotics in the tissue was too low to quantify in some cases.

### Summary.

All patients in this study received standard oral antimicrobial therapy treatment for their DFI, as described in local clinical guidelines. The bacterial load in tissue samples taken pre- and posttreatment, while statistically significant, was not profound. Unfortunately, ethical consent to provide clinical outcome information on these patients was not sought, so conclusions as to whether this statistically significant population reduction correlates with successful clinical outcome cannot be made.

However, we utilized an *in vitro* collagen model of a diabetic foot ulcer to simulate these patient specific DFIs. The microbiota taken from each patient was added to the model, and systemic therapy, aligned to that which they received from the clinic, at levels equivalent to their serum levels were added to the model. In parallel, vancomycin/gentamicin-loaded SRC beads were also added to the models. The vancomycin- and gentamicin-loaded SRC beads elicited a greater log reduction in bacterial load in all patient models compared to systemic therapy, which failed to significantly reduce bacterial load in all models.

Antibiotic-loaded SRC beads are a viable means of releasing high concentrations of antibiotics in close proximity to biofilm and is effective at significantly reducing biofilm bioburden within an *in vitro* model of a DFI. These data demonstrate that, in our *in vitro* model, antibiotic-loaded SRC beads perform better than current treatments prescribed for DFI. Moreover, this method when applied to the clinic may have the potential to avoid the need for high and/or extended antibiotic dosing regimens. The effects of this would be a reduction in adverse effects such as toxicity, a reduction in colonization resistance in the colon and the resultant risk of Clostridium difficile infection, the likelihood of the development of antimicrobial resistance, and reduced cost to health care systems.

## MATERIALS AND METHODS

### Ethics.

This study permitted the recruitment of up to 10 adult patients with type 1 or 2 diabetes whom were diagnosed with a DFI, with no prior treatment with oral/i.v. antibiotics or antimicrobial/antiseptic-containing dressings for at least 1 month prior to recruitment. Participants gave written informed consent to take part in the study.

Sponsorship approval for this study was obtained from University of Manchester by proportionate University Research Ethics Committee review. HRA and NHS REC approval was granted by Wales REC 6.

Patient recruitment took place at Whitworth and Buxton Hospitals, Derbyshire NHS Foundation Trust. Patient-identifiable information was anonymized by the podiatry team and was not available to the research team.

### Tissue and serum sample collection.

Wounds were debrided to remove devitalized tissue and surface-contaminating microbes, as per local practice. After this, samples were taken for microbiology analysis within the hospital, and remaining tissue was collected for the study (50). Tissue was stored on ice until collection by the research team for processing on the same day. Tissue samples were collected twice: pretreatment (upon diagnosis) and posttreatment (after 7 days of standard systemic antibiotic therapy).

Blood samples were collected from patients after 7 days of antibiotic therapy. Samples were maintained on ice, centrifuged at 13,000 × *g* for 7 min, and then stored at −80°C prior to antibiotic quantification.

Antibiotic prescribing information detailing the antimicrobial agent prescribed and the dosing frequency was collected for each patient, as well as the sex, the patient age at the time of infection, and whether or not the subject was obese, had PVD, or had neuropathy. Renal function, wound grade, adherence to antibiotic therapy, timing of the last dose, timing of sampling at day 7, and adherence to the antibiotic regimen were also recorded.

### Tissue sample processing.

Debrided tissue samples were weighed, phosphate-buffered saline (PBS; Sigma-Aldrich) was added, and each sample was then homogenized in a bead beater (Precellys 24 homogenizer) with 0.5-mm glass beads at 5,000 rpm for 5 s with 2-min rests between cycles until the tissue was completely homogenized. Samples were then centrifuged at 13,000 × *g* for 3 min, and the supernatant was stored at −80°C for subsequent antibiotic quantification (for tissue samples collected posttreatment only). The pellets were resuspended in PBS, centrifuged at 13,000 × *g* for 3 min, again resuspended and diluted 10-fold in PBS, and plated onto chocolate agar consisting of tryptone soy agar (TSA; Oxoid), supplemented with 10% defibrinated horse blood (VWR) before incubation at 37°C under aerobic and anaerobic conditions for 48 h.

Distinct bacterial colonies from plates incubated aerobically and anaerobically were enumerated and plated onto fresh chocolate agar; once established, pure bacterial colonies were transferred to 25% glycerol and frozen at −80°C for subsequent identification and antimicrobial susceptibility testing. This process was performed for both pre- and posttreatment tissue samples.

Bacterial growth from one chocolate agar dilution plate (for both aerobic and anaerobic incubation conditions), which was representative of the complete microbiome of the tissue sample, was removed and transferred to 25% glycerol and frozen at −80°C for subsequent inoculation into the collagen wound models. This was performed for pretreatment tissue samples.

### Identification of microorganisms.

Isolates were identified on the basis of colony morphology, Gram stain, biochemical reactivity, and 16S rRNA sequencing. For 16S rRNA sequencing, isolates from −80°C stocks were plated onto TSA, and a colony transferred to 20 μl of sterile MilliQ water, vortexed for 30 s, and boiled at 95°C for 10 min to lyse the cells. PCR was performed on the lysed cells using 16S rRNA primers 806R and 8F1P1 (51) and/or the standard Sigma primers 27F and 1492R (52). All primers were purchased from Sigma. The PCR cycle was run for 30 cycles, and PCR products were purified using a QIAquick PCR purification kit (Qiagen) according to the manufacturer’s instructions. The DNA yield was quantified by using a NanoDrop 2000 UV-vis spectrophotometer. A final reaction mixture comprising 20 to 50 ng of PCR product and 4 pmol of forward or reverse primer was used for DNA sequencing on an Applied Biosystems 3730 DNA analysis system at the DNA sequencing facility at the University of Manchester.

### Collagen wound models. (i) Preparation of wound models.

*In vitro* collagen wound models were prepared as described previously (37). The model described here incorporates human dermal fibroblasts into a collagen matrix. The models were constructed within a six-well plate (clear polystyrene, flat bottom, TC treated; Costar; Corning) and cell culture insert (3.0-μm pore size, transparent PET membrane; Falcon). A thin acellular collagen layer anchors a thicker cellular collagen matrix.

Human dermal fibroblasts, neonatal (HDFn; Gibco) were maintained according to the manufacturer’s guidelines, except that fibroblast growth medium (FGM) was used to maintain cell growth. FGM contained Dulbecco modified Eagle medium (with 1,000 mg/liter glucose and sodium bicarbonate, sterile-filtered; Sigma-Aldrich) supplemented with 8 mM HEPES (MP Biomedicals, France), 10% fetal bovine serum (Sigma-Aldrich), and 2 mM l-glutamine (Sigma-Aldrich).

An acellular collagen layer was prepared by diluting collagen (Rat Tail, 100 mg, 9.33 mg/ml; Corning) to 4 mg/ml in FGM and adjusting the pH to 7 to 7.5 using 7.5% sodium bicarbonate (Gibco). A 1-ml portion of acellular collagen mixture was added to the base of the cell culture insert and allowed to polymerize at room temperature.

A cellular collagen mixture was prepared by preparing stock solutions of type 1 collagen at 5 mg/ml (pH adjusted to 7 to 7.5), 8 mg/ml extracellular matrix proteins (ECM; Sigma-Aldrich), 10 mg/ml hyaluronic acid (Alfa Aesar), and 3 × 10^5^ cells/ml HDFn. These stock solutions were combined to give a final concentration of 4 mg/ml type 1 collagen, 75 μg/ml ECM, 1 mg/ml hyaluronic acid, and 2.34 × 10^4^ cells/ml HDFn. FGM was used as a diluent. Then, 6 ml of cellular collagen was added to each acellular layer, and a mold was fitted to the top of the six-well plate to create a void in the center of the collagen models 12 mm in diameter and 10 mm deep (36). The collagen mixture was allowed to polymerize at 37°C in 5% CO_2_ for 1 h. The model was removed, 3 ml of FGM was added to each well, and 1 ml of FGM was added to the surface of each model. Models were incubated at 37°C in 5% CO_2_ for 5 to 7 days to allow for HDFn-initiated collagen contraction. All collagen manipulations were performed on ice to prevent premature polymerization.

### (ii) Inoculation of models.

Frozen glycerol stocks of the patient microbiome were emulsified directly into PBS and diluted 10-fold in PBS to 10^−7^, and then 100 μl was inoculated onto chocolate agar, followed by incubation at 37°C for 24 h. The agar plate with a representative bacterial mix and between 50 and 100 colonies was identified, 1 ml of PBS was added to the plate and emulsified, and the bacterial emulsion was then transferred to an Eppendorf containing 1 ml of PBS. This emulsion was standardized to an optical density at 600 nm (OD_600_) of 1 in PBS before dilution to 1:1,000 in PBS. Models were inoculated with 100 μl of this standardized bacterial emulsion plus 300 μl of sterile FGM, followed by incubation at 37°C in 5% CO_2_ for 3 days.

### (iii) Addition of antibiotics.

Antibiotic-loaded calcium sulfate (CS) beads were prepared by combining 10 ml of CS (Stimulan Rapid Cure, Biocomposites, Ltd.) with three 2-ml vials of 80 mg/ml gentamicin (240 mg in total) and 500 mg of vancomycin, with thorough mixing to form a paste before being pressed into 3-mm-diameter hemispherical cavities in a flexible mold. The beads were allowed to cure prior to removal from the mold.

To the void in the collagen wound model we added either (i) 400 mg of vancomycin- and gentamicin-loaded CS beads (500 mg of vancomycin, 240 mg of gentamicin, 10 ml of CS) such that they filled the space, (ii) simulated systemic antibiotics (at levels equal to the serum levels for each patient or the *C*_max_ from the literature, where the serum levels were not available), or (iii) no antibiotics (FGM only), followed by incubation at 37°C in 5% CO_2_ for 3 days.

### (iv) Bacterial quantification.

Models were removed from the cell culture inserts, and then sections of roughly 5-mm^3^ collagen were sliced and transferred to an Eppendorf. The weight of the section was then determined. A volume of collagenase (type 1; MP Biomedicals) equal to the weight of the section was added, vortexed, and incubated at 37°C until dissolved. Sections were then centrifuged at 13,000 × *g* for 3 min twice. Bacteria were pelleted, and supernatants were stored at −80°C until transport to the Antimicrobial Reference Laboratory for antibiotic quantification (Southmead Hospital, North Bristol NHS Trust, UK). Bacterial pellets were resuspended in PBS, diluted, plated onto TSA in triplicate, and incubated aerobically at 37°C for 24 to 48 h. Colonies were counted, and log reductions were calculated and compared to no-antibiotic control models. All *P* values were determined by using a paired Student *t* test.

### (v) Quantification of antibiotics.

In order to determine the antibiotic levels to apply to the inoculated collagen wound mode, the levels of the antibiotics prescribed to the study subjects were determined in blood and tissue. The levels of amoxicillin, sulfamethoxazole, trimethoprim, and ciprofloxacin were measured using validated assays. For the first three analytes, these were based on liquid chromatography (LC) with UV detection and had LLOQs of 1 mg/liter. The assay for ciprofloxacin was based on LC with fluorescence detection and had an LLOQ of 0.1 mg/liter. Flucloxacillin was quantified using an in-house method based on LC-MS/MS, with an LLOQ of 0.1 mg/liter. All the assays used protein precipitation for extracting the analytes, methanol for ciprofloxacin, and acetonitrile for the rest.

### MICs.

The MICs of the antibiotic systemically administered to each patient (as part of their prescribed treatment) were determined on isolates recovered from pre- and posttreatment tissue samples. The MICs of vancomycin (500 mg)-gentamicin (240 mg) combinations were also determined on isolates recovered from pretreatment tissue samples.

Isolates were cultured onto TSA from glycerol freezer stocks. Broth microdilution MICs were performed in TSB with antibiotic serial dilutions prepared in PBS and sterilized by filtrations through 0.22-μm syringe filters (PES, Millex-GP; Millipore). Bacterial cultures equal to an OD_600_ of 1.0 were diluted 1:50 in double-concentrated TSB. The cultures were then added to antibiotic serial dilutions within a 96-well microplate at a ratio of 1:1, resulting in a 2-fold dilution of antibiotic solutions. Microplates were incubated at 37°C for 24 h. MICs were determined as the lowest concentration of antibiotic required to inhibit bacterial growth as determined by measuring the OD_600_ on a SpectraStar nanoplate reader. Assays were performed with eight technical and three biological repeats.

### Data availability.

All data generated or analyzed during this study are included in this published article.
